# ﻿Two new oonopid spiders (Arachnida, Araneae) from Xishuangbanna tropical rainforest, Yunnan, China

**DOI:** 10.3897/zookeys.1205.124183

**Published:** 2024-07-01

**Authors:** Yanfeng Tong, Yongbo Shao, Dongju Bian, Shuqiang Li

**Affiliations:** 1 College of Life Science, Shenyang Normal University, Shenyang 110034, China Shenyang Normal University Shenyang China; 2 Key Laboratory of Forest Ecology and Management, Institute of Applied Ecology, Chinese Academy of Sciences, Shenyang 110016, China Institute of Applied Ecology, Chinese Academy of Sciences Shenyang China; 3 Institute of Zoology, Chinese Academy of Sciences, Beijing 100101, China Institute of Zoology, Chinese Academy of Sciences Beijing China

**Keywords:** Biodiversity, goblin spiders, taxonomy

## Abstract

A new species of the genus *Bannana* Tong & Li, 2015 and a new species of the genus *Trilacuna* Tong & Li, 2007 are recorded from Xishuangbanna, Yunnan Province: *Bannanazhengguoi* Tong & Li, **sp. nov.** (♂♀) and *Trilacunaaoxian* Tong & Li, **sp. nov.** (♂♀). An identification key to species of the genus *Bannana* from Xishuangbanna is provided. Detailed diagnoses, descriptions, and photomicroscopy images of new species are provided.

## ﻿Introduction

Goblin spiders (Araneae, Oonopidae) are small (usually <3 mm), six-eyed, haplogyne, non-web-building spiders. They have a nearly worldwide distribution and occur mainly in leaf litter, under bark, and in the tree canopy (Jocqué and Dippenaar-Schoeman 2006; Ubick and Dupérré 2017). Currently, 1952 extant described species in 115 genera of oonopid spiders have been recorded in the world, in which 17 genera and 118 species are distributed in China (WSC 2024).

Xishuangbanna, located in southern Yunnan Province, has the best-preserved tropical rainforest in China and belongs to the Indo-Burma biodiversity hotspot (Myers 1988). The survey of oonopid spiders from Xishuangbanna has started relatively recently. To date, 28 species in five genera have been recorded from Xishuangbanna (Huang et al. 2021; Tong et al. 2021; Song et al. 2024).

The genus *Bannana* was established by Tong and Li (2015). Only three species have been described, *B.crassispina* Tong & Li, 2015, *B.parvula* Tong & Li, 2015, and *B.songxiaobini* Tong & Li, 2019. All three species are endemic to Xishuangbanna, Yunnan (Tong and Li 2015; Sun et al. 2019). The genus *Trilacuna* Tong & Li, 2007 currently comprised 43 species. All species are known from Iran to the Korean Peninsula and south to Sumatra (Tong and Li 2007; Eichenberger and Kranz-Baltensperger 2011; Grismado et al. 2014; Malek-Hosseini et al. 2015). In China, the genus is represented by 21 species, of which 12 species are known in Yunnan Province (Tong et al. 2019; Ma et al. 2023). There are no distribution records of this genus in Xishuangbanna until now. The present paper describes two new species of *Bannana* and *Trilacuna* from this region.

## ﻿Materials and methods

All the specimens used in this study were collected by pitfall trapping or searching by hand in forest leaf litter and later examined using a Leica M205C stereomicroscope. Details of body parts and measurements were studied under an Olympus BX51 compound microscope. Photos were made with a Canon EOS 750D zoom digital camera (18 megapixels) mounted on an Olympus BX51 compound microscope. Left palps were detached for taking images. Endogyne were cleared in 85.0–90.0% lactic acid at normal temperature. Scanning electron microscope images (SEM) were taken under high vacuum with a Hitachi TM3030 after critical-point drying and gold-palladium coating. All measurements in the text are expressed in millimeters. All materials studied are deposited in Shenyang Normal University (**SYNU**) in Shenyang, China.

Taxonomic descriptions follow Tong and Li (2015) and Tong et al. (2020). The following abbreviations are used in the text and figures: **ALE** = anterior lateral eyes; **ALE–PLE** = distance between ALE and PLE; **ap** = apodemes; **as** = anterior sclerite; **boc** = booklung covers; **cls** = comb-like structure; **cmp** = clypeus median projection; **db** = dorsal branch; **dep** = deep depressions; **emb** = embolus; **glo** = tube-like globular structure; **ldi** = labium deep incision; **pb** = posterior branch; **PLE** = posterior lateral eyes; **PME** = posterior median eyes; **se** = serrula; **spb** = slender posterior branch; **tba** = transverse bars; **tp** = triangular plate; **tsc** = transverse sclerite; **vb** = ventral branch; **XNNR** = Xishuangbanna National Natural Reserve; **XTBG** = Xishuangbanna Tropical Botanical Garden.

## ﻿Taxonomy

### ﻿Family Oonopidae Simon, 1890

#### ﻿Genus Bannana

Taxon classificationAnimaliaAraneaeOonopidae

Tong & Li, 2015

3288B28B-A4F1-5EC0-A695-F25C7D6497D6

##### Type species.

*Bannanacrassispina* Tong & Li, 2015; gender feminine.

### ﻿Key to species of *Bannana* from Xishuangbanna, China

Male of *B.songxiaobini* is unknown.

**Table:** 

1	Male	**2**
–	Female	**4**
2	Eyes not reduced (Fig. 1B, D); with group of thick setae on epigastric region and short comb-like structure on dorsal branch of bulb (Figs 1G, 4F)	***B.zhengguoi* sp. nov.**
–	Without the aforementioned character	**3**
3	With rows of setae on the central part of sternum and thick bristles on palpal tibiae (Tong and Li 2015: figs 1C, 2A, D)	***B.crassispina* Tong & Li, 2015**
–	Without aforementioned characters	***B.parvula* Tong & Li, 2015**
4	Eyes not reduced (Fig. 2B, G); posterior spiracles not connected by groove (Figs 2H, 3D)	***B.zhengguoi* sp. nov.**
–	Eyes reduced (Tong and Li 2015: figs 3E, D, 5A, D); posterior spiracles connected by groove (Tong and Li 2015: fig. 6D, E)	**5**
5	Postepigastric scutum long, nearly quadrangular, the distance between the groove connected posterior spiracles to posterior margin nearly three times the distance to anterior margin (Sun et al. 2019: fig. 1G)	***B.songxiaobini* Tong & Li, 2019**
–	Postepigastric scutum short, only around epigastric furrow, the distance between the groove connected posterior spiracles to posterior margin nearly equal to or shorter than the distance to anterior margin (Tong and Li 2015: figs 3G, 5G)	**6**
6	Dorsal scutum covering about 5/6 of abdomen, about equal to the abdomen width (Tong and Li 2015: fig. 5E); the distance between the groove connected posterior spiracles to posterior margin nearly equal to the distance to anterior margin (Tong and Li 2015: figs 5I, 6D)	***B.parvula* Tong & Li, 2015**
–	Dorsal scutum covering about 3/4 of abdomen, about 2/3 of abdomen width (Tong and Li 2015: fig. 3A); the distance between the groove connected posterior spiracles to posterior margin nearly equal to half the distance to anterior margin (Tong and Li 2015: figs 2H, 3H)	***B.crassispina* Tong & Li, 2015**

#### ﻿ Bannana zhengguoi

Taxon classificationAnimaliaAraneaeOonopidae

Tong & Li sp. nov.

7F2569B3-903D-5863-AFCD-112FE144B6A2

https://zoobank.org/37E10DC8-C19D-4237-830B-FE6C2DA9E78E

Figs 1 , 2 , 3 , 4

##### Type materials.

***Holotype*** ♂ (SYNU-1051): China, Yunnan Prov., Menglun, XTBG, primary tropical seasonal rain forest, searching by hand, 21°55.035'N, 101°16.500'E, 558 m, Guo Zheng leg., 4–11/5/2007. ***Paratypes***: 1♂1♀ (SYNU-1052–1053), same data as holotype; 1♂ (SYNU-1040), XNNR, secondary tropical montane evergreen broad-leaved forest, pitfall traps, 21°54.767'N, 101°11.431'E, 880 m, Guo Zheng leg., 16–31/4/2007; 1♀ (SYNU-1037), XTBG, primary tropical seasonal rain forest, searching by hand, 21°55.035'N, 101°16.500'E, 558 m, Guo Zheng leg., 4–11/5/2007; 1♂ (SYNU-1038), XNNR, primary tropical seasonal rain forest, pitfall traps 21°57.669'N, 101°11.893'E, 790 m, Guo Zheng leg., 1–15/4/2007; 1♀ (SYNU-1039), XTBG, primary tropical seasonal rain forest, searching by hand, 21°55.035'N, 101°16.500'E, 558 m, Guo Zheng leg., 19–26/5/2007; 3♂ (SYNU-1041–1043), XNNR, primary tropical seasonal rain forest, pitfall traps, 21°57.669'N, 101°11.893'E, 790 m, Guo Zheng leg., 1–15/5/2007; 1♀ (SYNU-1044), XNNR, secondary tropical seasonal moist forest, searching by hand, 21°54.607'N, 101°17.005'E, 633 m, Guo Zheng leg., 19–26/5/2007; 1♀ (SYNU-1045), XTBG, primary tropical seasonal rain forest, searching by hand, 21°55.035'N, 101°16.500'E, 558 m, Guo Zheng leg., 1–15/7/2007; 1♂ (SYNU-1046), XTBG, primary tropical seasonal rain forest, pitfall traps, 21°55.035'N, 101°16.500'E, 558 m, Guo Zheng leg., 16–31/4/2007; 1♂ (SYNU-1047), XTBG, rubber-tea plantation (about 20 yr.), pitfall traps, 21°55.551'N, 101°16.923'E, 561 m, Guo Zheng leg., 16–31/6/2007.

##### Diagnosis.

**Figure 1.**
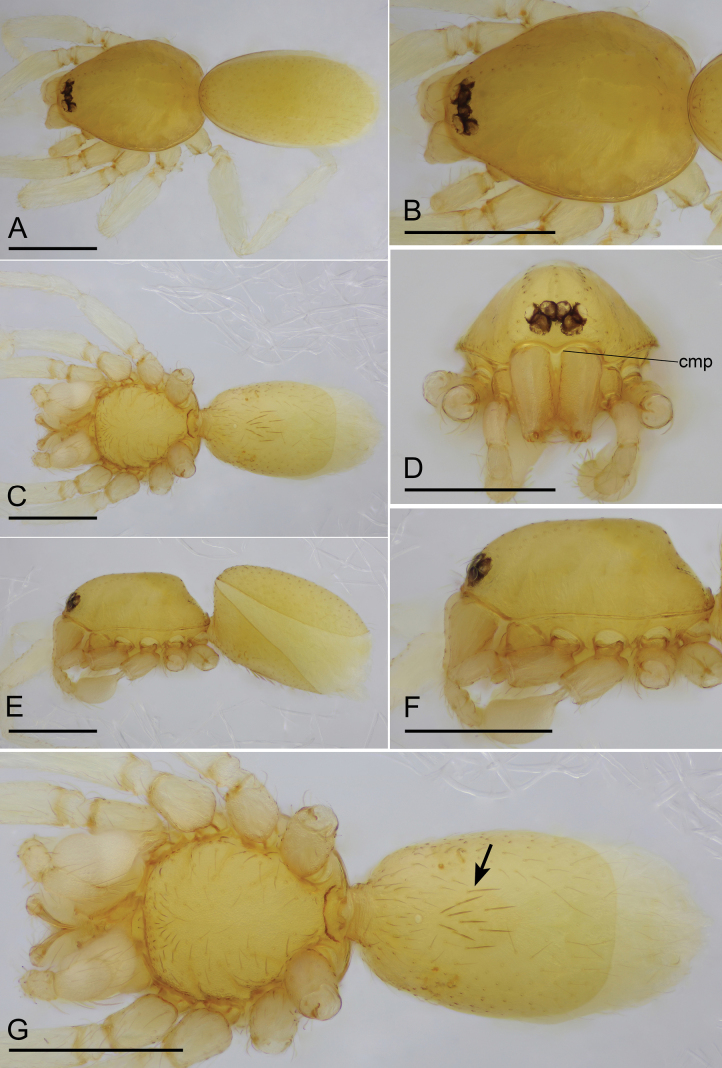
*Bannanazhengguoi* sp. nov., male holotype **A, C, E** habitus in dorsal, ventral, and lateral views **B, D, F** prosoma in dorsal, anterior and lateral views **G** habitus in ventral view, black arrow shows the group of thick setae. Abbreviation: cmp = clypeus median projection. Scale bars: 0.4 mm (**A–G**).

**Figure 2.**
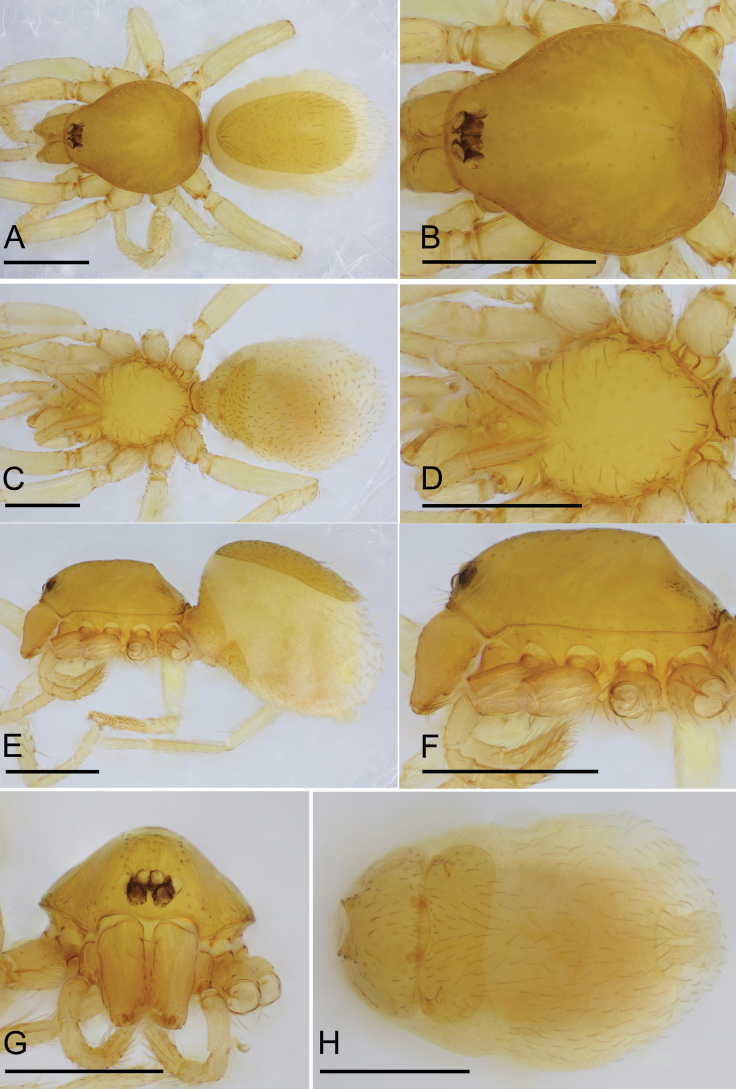
*Bannanazhengguoi* sp. nov., female paratype (SYNU-1053) **A, C, E** habitus in dorsal, ventral, and lateral views **B, D, F, G** prosoma in dorsal, ventral, lateral and anterior views **H** abdomen in ventral view. Scale bars: 0.4 mm (**A–H**).

**Figure 3.**
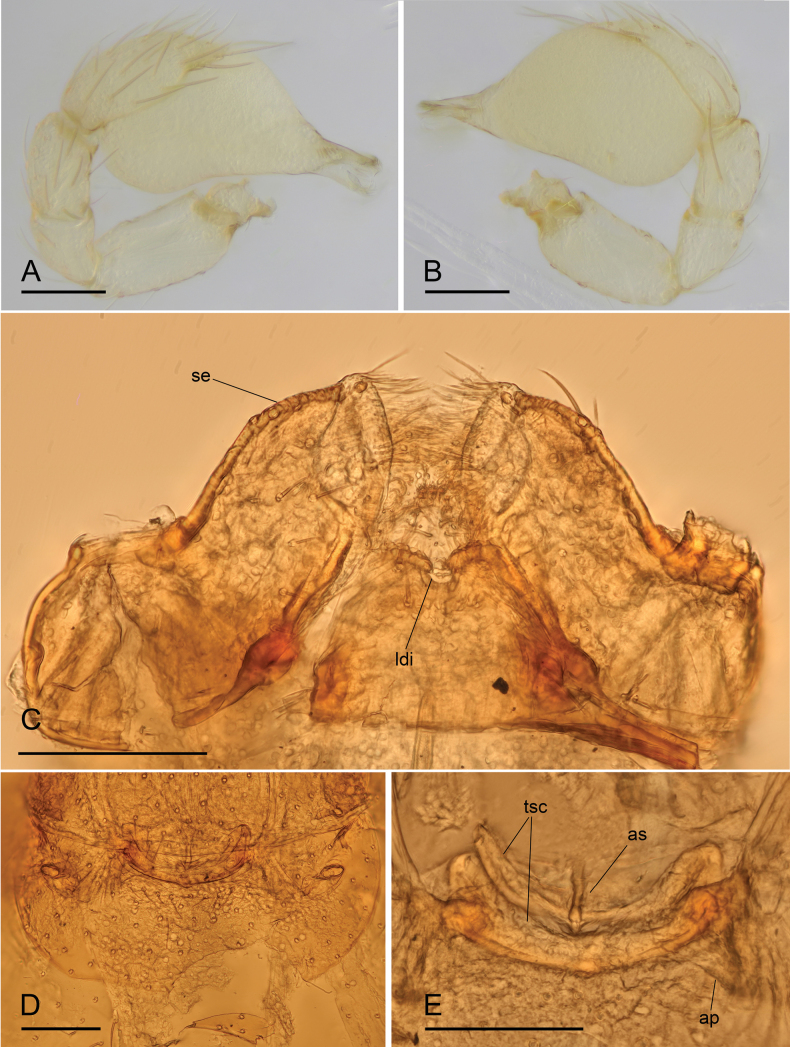
*Bannanazhengguoi* sp. nov. **A** male left palp, prolateral view **B** male left palp, retrolateral view **C** male endites and labium, ventral view **D, E** endogyne in ventral and dorsal views. Abbreviations: ap = apodemes; as = anterior sclerite; ldi = labium deep incision; se = serrula; tsc = transverse sclerite. Scale bars: 0.1 mm (**A–E**).

**Figure 4.**
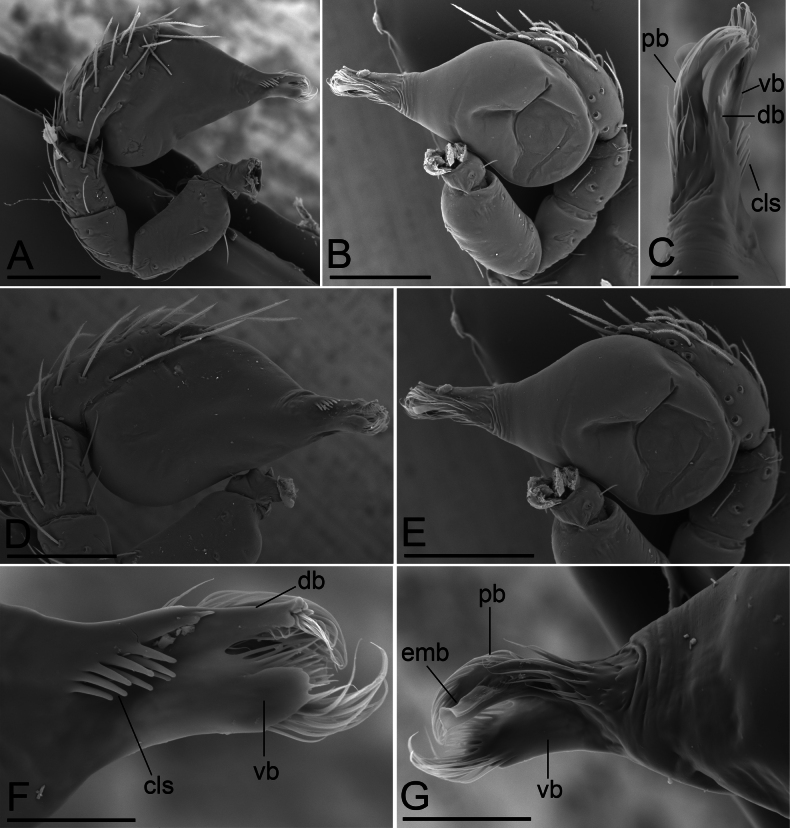
*Bannanazhengguoi* sp. nov., male left palp, SEM **A, B** prolateral and retrolateral views **C, F, G** distal part of bulb, dorsal, prolateral and retrolateral views **D, E** bulb, prolateral and retrolateral views. Abbreviations: cls = comb-like structure; db = dorsal branch; emb = embolus; pb = posterior branch; vb = ventral branch. Scale bars: 0.1 mm (**A, B, D, E**); 0.03 mm (**C, F, G**).

The new species can be distinguished from all the congeners in having the eyes not reduced (Figs 1D, 2G), vs reduced eyes (Tong and Li 2015: figs 1F, 3D, 4C, 5D; Sun et al. 2019: fig. 1H). Furthermore, males of the new species can be distinguished from those of *B.crassispina* and *B.parvula* by the short comb-like structure on dorsal branch of bulb and group of thick setae on epigastric region (Figs 1G, 4F), vs without comb-like structure and group of thick setae (Tong and Li 2015: figs 1G, 2E, 4F, 6A); females of the new species can be distinguished from those of *B.crassispina*, *B.parvula*, and *B.songxiaobini* by the posterior spiracles not connected by groove (Figs 2H, 3D), vs connected (Tong and Li 2015: figs 3G, H, I, 5G; Sun et al. 2019: fig. 1G).

##### Description.

**Male (holotype). *Body*** yellow, chelicerae, sternum, and legs lighter; habitus as in Fig. 1A, C, E; body length 1.45. ***Carapace*** (Fig. 1B, F): 0.68 long, 0.50 wide; pars cephalica almost flat in lateral view, surface smooth. ***Eyes*** (Fig. 1B, D): ALE largest; PLE and PME nearly equal in size; ALE–PLE separated by less than ALE radius; PME touching each other; posterior eye row recurved as viewed from above, straight as viewed from front. ***Clypeus*** (Fig. 1D): height about 0.5 times of ALE diameter, with a triangular clypeus median projection (cmp). ***Mouthparts*** (Fig. 3C): labium deeply incised. ***Sternum*** (Fig. 1G): surface finely reticulate. Abdomen (Fig. 1A, C, E, G): 0.82 long, 0.45 wide; dorsal scutum nearly covering full length of abdomen, postepigastric and epigastric scutum fused, covering 5/6 of abdomen length; book lung covers ovoid, surface smooth; epigastric region with a group of thick setae, posterior spiracles not connected by groove. ***Palp*** (Figs 3A, B, 4A–G): pale-orange; 0.48 long (0.15, 0.09, 0.08, 0.16); femur elongated (width/length = 0.48); bulb oval, tapering apically; embolus system complicated, with a cluster of short comb-like structures on dorsal branch, many hair-like structure on ventral and posterior branches.

**Female (SYNU-1053).** Same as male except as noted. ***Body*** habitus as in Fig. 2A, C, E; body length 1.51. ***Carapace*** (Fig. 2B, F): 0.66 long, 0.53 wide. ***Abdomen*** (Fig. 2A, C, E, H): 0.84 long, 0.56 wide; dorsal scutum covering about 4/5 of abdomen length, about 2/3 of abdomen width; postepigastric scutum rectangular, posterior margin nearly straight. ***Epigaster*** (Fig. 3D): surface without external features. ***Endogyne*** (Fig. 3E): with two narrow, transverse sclerites (tsc) and an anterior stick-shaped sclerite (as); lateral apodemes (ap) present.

##### Etymology.

The specific name is named in honor of the collector, Mr Guo Zheng.

##### Distribution.

Known only from the type locality.

#### ﻿Genus Trilacuna

Taxon classificationAnimaliaAraneaeOonopidae

Tong & Li, 2007

EDE38AB3-77C3-55AC-BDA9-47808CF57F45

##### Type species.

*Trilacunarastrum* Tong & Li, 2007; gender feminine.

#### ﻿ Trilacuna aoxian

Taxon classificationAnimaliaAraneaeOonopidae

Tong & Li sp. nov.

56544E8D-FF05-5591-BFB9-1664F04B49E3

https://zoobank.org/0E605FF2-B545-407D-B8AD-775025FD0E03

Figs 5 , 6 , 7 , 8

##### Type materials.

***Holotype*** ♂ (SYNU-989): China, Yunnan Prov., Menglun, XNNR, primary tropical seasonal rain forest, pitfall traps, 21°57.669'N, 101°11.893'E, 790 m, Guo Zheng leg.,16–31/3/2007; ***Paratypes***: 1♀ (SYNU-990), XTBG, primary tropical seasonal rain forest, searching by hand, 21°55.035'N, 101°16.500'E, 558 m, Guo Zheng leg., 5–12/1/2007; 1♀ (SYNU-991), XTBG, primary tropical seasonal rain forest, searching by hand, 21°55.035'N, 101°16.500'E, 558 m, Guo Zheng leg., 19–25/11/2006; 1♀ (SYNU-992), XTBG, primary tropical seasonal rain forest, searching by hand, 21°55.035'N, 101°16.500'E, 558 m, Guo Zheng leg., 5–12/12/2006; 1♂ (SYNU-993), XNNR, secondary tropical montane evergreen broad-leaved forest, pitfall traps, 21°54.767'N, 101°11.431'E, 880 m, Guo Zheng leg., 1–15/3/2007; 1♀ (SYNU-994), XNNR, secondary tropical seasonal moist forest, pitfall traps, 21°54.607'N, 101°17.005'E, 633 m, Guo Zheng leg., 16–31/6/2007; 1♀ (SYNU-995), XNNR, primary tropical seasonal rain forest, pitfall traps, 21°55.035'N, 101°16.500'E, 558 m, Guo Zheng leg., 1–15/4/2007; 4♂ (SYNU-996–999), XTBG, primary tropical seasonal rain forest, pitfall traps, 21°55.035'N, 101°16.500'E, 558 m, Guo Zheng leg., 1–15/1/2007; 3♂ (SYNU-1000–1002), XTBG, primary tropical seasonal rain forest, pitfall traps, 21°55.035'N, 101°16.500'E, 558 m, Guo Zheng leg., 16–31/2/2007; 1♂ (SYNU-1003), XNNR, primary tropical seasonal rain forest, pitfall traps, 21°57.445'N, 101°12.997'E, 744 m, Guo Zheng leg., 16–31/2/2007; 1♀ (SYNU-1004), XTBG, primary tropical seasonal rain forest, searching by hand, 21°55.035'N, 101°16.500'E, 558 m, Guo Zheng leg., 19–25/11/2006; 1♂ (SYNU-1005), XNNR, secondary tropical seasonal moist forest, pitfall traps, 21°54.607'N, 101°17.005'E, 633 m, Guo Zheng leg., 1–15/3/2007; 2♂1♀ (SYNU-1006–1008), XTBG, primary tropical seasonal rain forest, pitfall traps, 21°55.035'N, 101°16.500'E, 558 m, Guo Zheng leg., 16–31/1/2007; 1♂ (SYNU-1009), XNNR, primary tropical seasonal rain forest, pitfall traps, 21°57.669'N, 101°11.893'E, 790 m, Guo Zheng leg., 16–31/2/2007; 2♂1♀ (SYNU-1010–1012), XTBG, primary tropical seasonal rain forest, pitfall traps, 21°55.035'N, 101°16.500'E, 558 m, Guo Zheng leg., 1–15/1/2007; 1♂ (SYNU-1013), XNNR, secondary tropical seasonal moist forest, searching by hand, 21°54.607'N, 101°17.005'E, 633 m, Guo Zheng leg., 19–25/2/2007; 3♂ (SYNU-1014–1016), XNNR, primary tropical seasonal rain forest, pitfall traps, 21°57.445'N, 101°12.997'E, 744 m, Guo Zheng leg., 16–31/2/2007; 3♂ (SYNU-1017–1019), XNNR, primary tropical seasonal rain forest, pitfall traps, 21°57.669'N, 101°11.893'E, 790 m, Guo Zheng leg., 1–15/2/2007; 1♂ (SYNU-1023), XNNR, primary tropical seasonal rain forest, pitfall traps, 21°57.445'N, 101°12.997'E, 744 m, Guo Zheng leg., 16–31/2/2007; 1♀ (SYNU-1024), XNNR, secondary tropical seasonal moist forest, pitfall traps, 21°54.607'N, 101°17.005'E, 633 m, Guo Zheng leg., 1–15/7/2007; 1♀ (SYNU-1027), XNNR, primary tropical seasonal rain forest, searching by hand, 21°57.669'N, 101°11.893'E, 790 m, Guo Zheng leg., 19–25/10/2006; 1♂ (SYNU-1028), XNNR, primary tropical seasonal rain forest, searching by hand, 21°57.445'N, 101°12.997'E, 744 m, Guo Zheng leg., 19–25/1/2007; 1♂ (SYNU-1029), XTBG, primary tropical seasonal rain forest, pitfall traps, 21°55.035'N, 101°16.500'E, 558 m, Guo Zheng leg., 16–31/2/2007; 1♀ (SYNU-1030), XTBG, primary tropical seasonal rain forest, pitfall traps, 21°55.035'N, 101°16.500'E, 558 m, Guo Zheng leg., 16–31/2/2007; 1♀ (SYNU-1031), XNNR, primary tropical seasonal rain forest, searching by hand, 21°57.669'N, 101°11.893'E, 790 m, Guo Zheng leg., 19–25/2/2007; 1♂ (SYNU-1032), XNNR, primary tropical seasonal rain forest, pitfall traps, 21°57.669N, 101°11.893'E, 790 m, Guo Zheng leg., 1–15/1/2007; 1♀ (SYNU-1033), XNNR, primary tropical seasonal rain forest, searching by hand, 21°57.445'N, 101°12.997'E, 744 m, Guo Zheng leg., 10–20/6/2007; 3♂ (SYNU-1034–1036), XNNR, primary tropical seasonal rain forest, pitfall traps, 21°57.669'N, 101°11.893'E, 790 m, Guo Zheng leg., 16–31/2/2007; 2♂1♀ (SYNU-1048–1050), XTBG, primary tropical seasonal rain forest, pitfall traps, 21°55.035'N, 101°16.500'E, 558 m, Guo Zheng leg., 1–15/1/2007.

##### Diagnosis.

**Figure 5.**
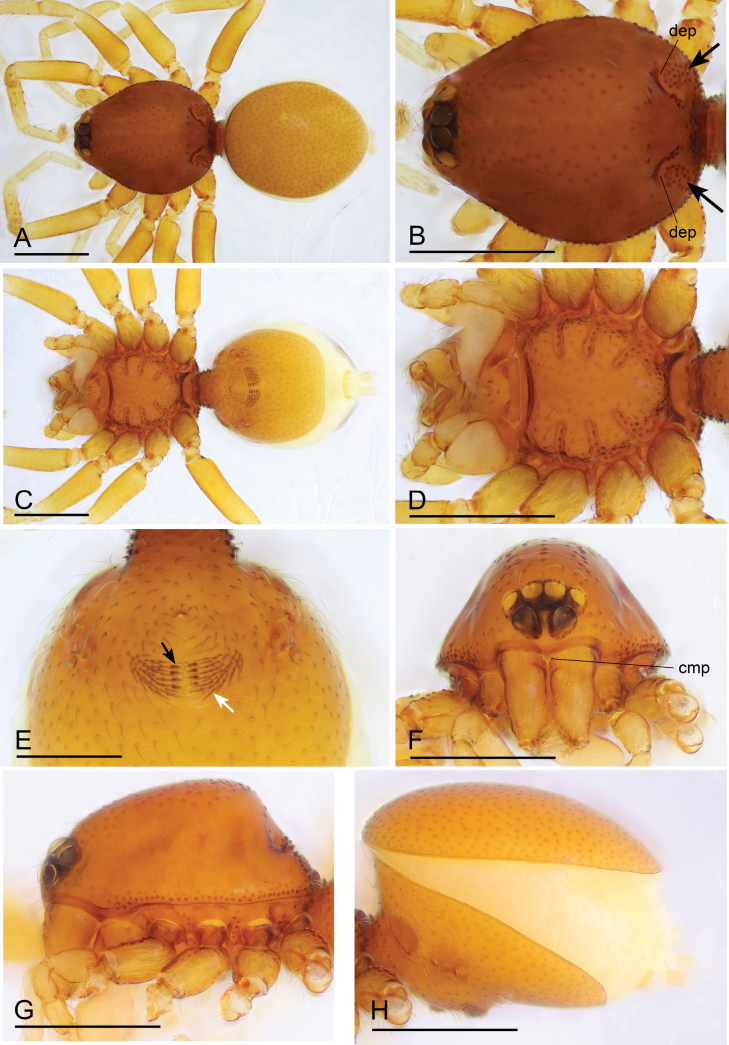
*Trilacunaaoxian* sp. nov., male holotype **A, C** habitus in dorsal and ventral views **B, D, F, G** prosoma in dorsal, ventral, anterior and lateral views, black arrows show large hair bases **E, H** abdomen in ventral and lateral views, black arrow shows the rows of short, black thorn-like setae, white arrow shows cluster of short setae. Abbreviations: cmp = clypeus median projection; dep = deep depressions. Scale bars: 0.4 mm (**A–D, F–H**); 0.2 mm (**E**).

**Figure 6.**
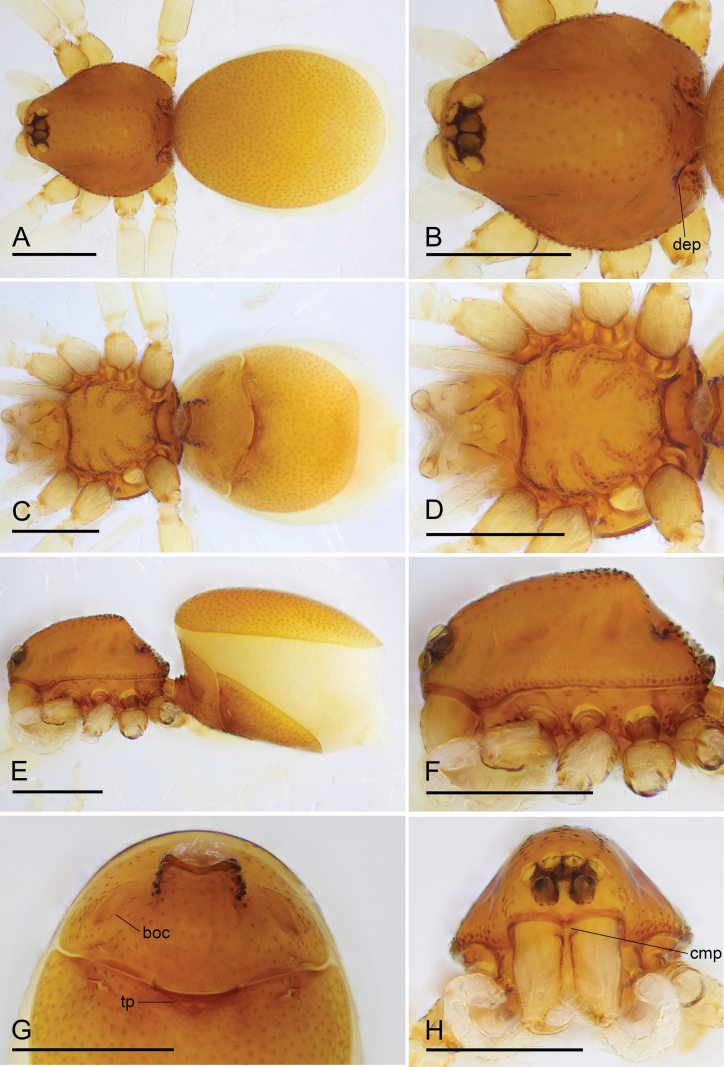
*Trilacunaaoxian* sp. nov., female paratype (SYNU-990) **A, C, E** habitus in dorsal, ventral, and lateral views **B, D, F, H** prosoma in dorsal, ventral, lateral and anterior views **G** abdomen in ventral view. Abbreviations: boc = booklung covers; cmp = clypeus median projection; dep = deep depressions; tp = triangular plate. Scale bars: 0.4 mm (**A–H**).

The new species is similar to *Trilacunachangzi* Tong & Li, 2020 in having long, thick setae on male endites, the tortuous, tube-like globular structure of endogyne, and the deep depressions on carapace, but it can be distinguished by the densely mucronate structure on sub-distal area of bulb (Fig. 8F, H) vs without mucronate structure (Tong et al. 2020: figs 5A, C, E), the rows of short, black thorn-like setae and cluster of short setae on epigastric region of male (Fig. 5E) vs a cluster of dense, short setae behind epigastric region (Tong et al. 2020: arrow in fig. 4C), and the triangular plate on epigastric region of female having the height/length = 0.33 (Fig. 6G), vs the height/length = 0.45 in *T.changzi* (Tong et al. 2020: fig. 6G).

##### Description.

**Figure 7.**
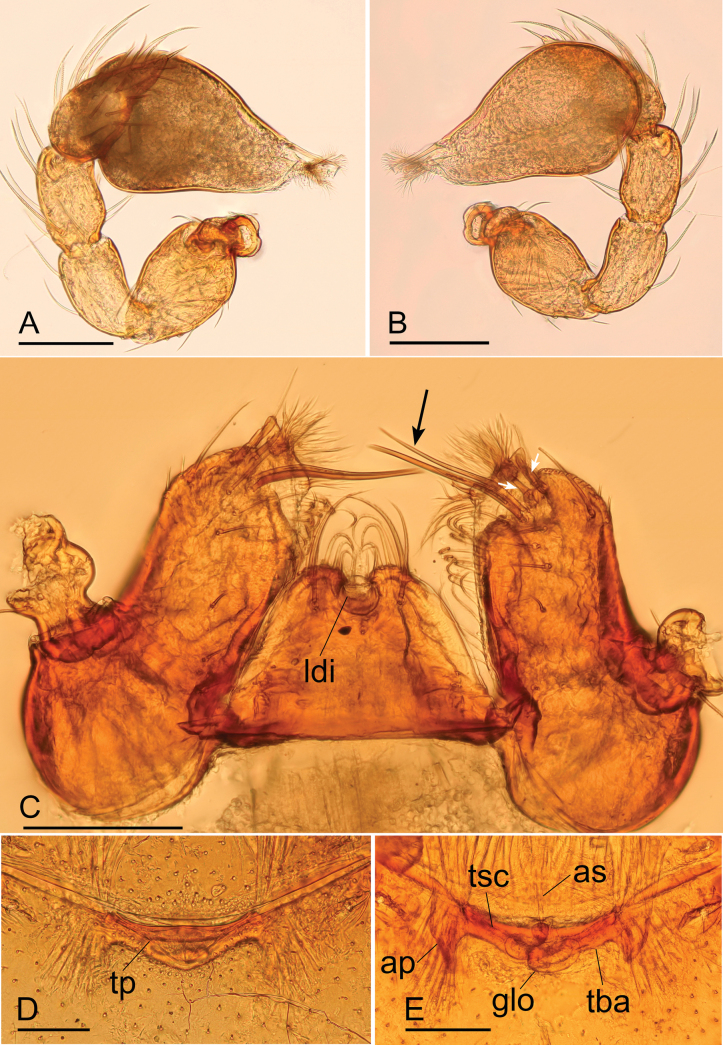
*Trilacunaaoxian* sp. nov. **A** male left palp, prolateral view **B** male left palp, retrolateral view **C** male endites and labium, ventral view, white arrow shows the short, thick setae, black arrow shows two very long, thick setae **D, E** endogyne in ventral and dorsal views. Abbreviations: ap = apodemes; as = anterior sclerite; glo = tube-like globular structure; ldi = labium deep incision; tba = transverse bars; tp = triangular plate; tsc = transverse sclerite. Scale bars: 0.1 mm (**A–E**).

**Figure 8.**
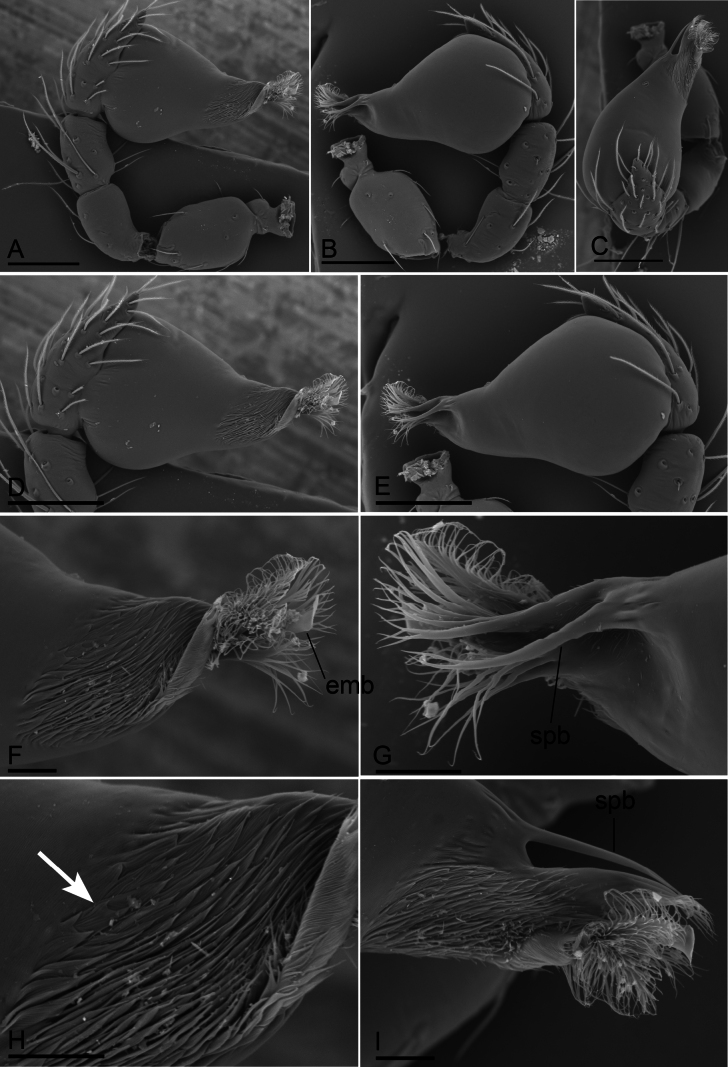
*Trilacunaaoxian* sp. nov., male left palp, SEM **A, B, C** prolateral, retrolateral and dorsal views **D, E** bulb, prolateral and retrolateral views **F, G, I** distal part of bulb, prolateral, retrolateral and dorsal views **H** detail of bulb, arrow shows the mucronate structure. Abbreviations: emb = embolus; spb = slender posterior branch. Scale bars: 0.1 mm (**A–E**); 0.02 mm (**F–I**).

**Male (holotype). *Body*** yellowish brown, chelicerae, sternum and legs lighter; habitus as in Fig. 5A, C; body length 1.62. ***Carapace*** (Fig. 5B, G): 0.77 long, 0.63 wide; sides smooth; lateral margin with small denticles; posterior surface with deep depressions and group of large hair bases. ***Eyes*** (Fig. 5B, F): ALE largest; PLE and PME nearly equal in size; ALE–PLE separated by less than ALE radius; PME touching each other; posterior eye row recurved as viewed from above, procurved as viewed from front. ***Clypeus*** (Fig. 5F): height about 0.75 times of ALE diameter, with a triangular, pointed, clypeus median projection (cmp). ***Mouthparts*** (Fig. 7C): labium deeply incised, endites with two short, thick setae and two very long, thick setae. ***Sternum*** (Fig. 5D): surface smooth. ***Abdomen*** (Fig. 5A, C, E, H): 0.86 long, 0.66 wide; booklung covers ovoid, surface smooth; epigastric region strongly elevated, with two rows of short, black thorn-like setae and cluster of short setae. ***Palp*** (Figs 7A, B, 8A–I): orange; 0.57 long (0.15, 0.13, 0.11, 0.18); femur elongated (width/length = 0.64); bulb oval, with densely mucronate structure on sub-distal area; embolus system with many hair-like structure and a single slender posterior branch.

**Female (SYNU-990).** Same as male except as noted. ***Body*** habitus as in Fig. 6A, C, E; body length 1.81. ***Carapace*** (Fig. 6B, F): 0.73 long, 0.65 wide. ***Abdomen*** (Fig. 6A, C, E, G): 1.08 long, 0.82 wide. ***Epigaster*** (Figs 6G, 7D): with a triangular plate, the height/length = 0.33. ***Endogyne*** (Fig. 7E): with narrow, transverse sclerite (tsc), an anterior stick-shaped sclerite (as), and a posterior tortuous, tube-like globular structure (glo); transverse bars (tba) with two lateral apodemes (ap).

##### Etymology.

The specific name comes from Chinese pinyin, “aoxian”, which means “depression” and is in reference to the deep depression on posterior surface of carapace; noun in apposition.

##### Distribution.

Known only from the type locality.

## Supplementary Material

XML Treatment for Bannana

XML Treatment for Bannana zhengguoi

XML Treatment for Trilacuna

XML Treatment for Trilacuna aoxian

## ﻿Additional information

### Author ORCIDs

Yanfeng Tong https://orcid.org/0000-0002-4348-7029

Yongbo Shao https://orcid.org/0009-0005-1156-6725

Dongju Bian https://orcid.org/0000-0002-9324-8026

Shuqiang Li https://orcid.org/0000-0002-3290-5416

### Data availability

All of the data that support the findings of this study are available in the main text.
